# Deep learning-based segmentation of subcellular organelles in high-resolution phase-contrast images

**DOI:** 10.1247/csf.24036

**Published:** 2024-07-31

**Authors:** Kentaro Shimasaki, Yuko Okemoto-Nakamura, Kyoko Saito, Masayoshi Fukasawa, Kaoru Katoh, Kentaro Hanada

**Affiliations:** 1 Department of Biochemistry and Cell Biology, National Institute of Infectious Diseases, Shinjuku-ku, Tokyo 162-8640, Japan; 2 Biomedical Research Institute, National Institute of Advanced Industrial Science and Technology (AIST), Tsukuba-shi, Ibaragi 305-8566, Japan; 3 AIRC, National Institute of Advanced Industrial Science and Technology (AIST), Koto-ku, Tokyo 135-0064, Japan; 4 Center for Quality Management Systems, National Institute of Infectious Diseases, Shinjuku-ku, Tokyo 162-8640, Japan

**Keywords:** label-free imaging, organelle dynamics, apodized phase contrast, deep learning-based segmentation

## Abstract

Although quantitative analysis of biological images demands precise extraction of specific organelles or cells, it remains challenging in broad-field grayscale images, where traditional thresholding methods have been hampered due to complex image features. Nevertheless, rapidly growing artificial intelligence technology is overcoming obstacles. We previously reported the fine-tuned apodized phase-contrast microscopy system to capture high-resolution, label-free images of organelle dynamics in unstained living cells (Shimasaki, K. *et al.* (2024). Cell Struct. Funct., 49: 21–29). We here showed machine learning-based segmentation models for subcellular targeted objects in phase-contrast images using fluorescent markers as origins of ground truth masks. This method enables accurate segmentation of organelles in high-resolution phase-contrast images, providing a practical framework for studying cellular dynamics in unstained living cells.

## Introduction

Quantitative analysis of biological images requires extracting the regions of targeted objects (organelles or individual cells) from images. Thresholding methods based on brightness histograms, such as Otsu’s method (Otsu, 1979), are popular approaches for image segmentation. However, in broad-field grayscale images like electron microscopic and label-free optical microscopic images, these methods often fail because the objects are influenced not only by their brightness but also by other image features, including local patterns or textures. Recent advances in artificial intelligence (AI) technology, particularly deep learning (DL), have made the segmentation of specific areas from these images more feasible for life scientists (Ounkomol *et al.*, 2018; Ronneberger *et al.*, 2015). Establishing DL-based segmentation models with supervised training requires ground truth mask images, which are labeled images where each pixel is assigned the correct categories, serving as a reference or standard for training and evaluating models. Ground truth masks are often prepared manually by human experts based on heuristic criteria. This manual annotation process is burdensome and time-consuming, and training DL models on these subjective annotations can result in unstable and biased models (Segebarth *et al.*, 2020; Von Chamier *et al.*, 2021).

We recently reported a tuned-up apodized phase-contrast (APC) microscopy system to visualize the dynamic behaviors of various organelles simultaneously within unstained cells (Shimasaki *et al.*, 2024). This system can capture APC and fluorescent images near-simultaneously, obtaining the paired images with minimal spatiotemporal loss in living cells. To develop a method for quantitative analysis of phase-contrast subcellular images, we here leveraged machine learning (ML) technologies with the fine-tuned APC microscopy system to establish segmentation models for various organelles, exploiting fluorescent organelle marker images as the origins of ground truth masks of training data for deep learning. We provide procedures to separate targeted organelles in high-resolution phase-contrast images using publically available ML tools, establishing an analysis framework for studying organelle dynamics in unstained living cells.

## Materials and Methods

### Cell culture

The Vero cell line derived from African green monkey kidneys (JCRB9013) was cultured in Eagle’s minimum essential medium (EMEM) supplemented with 5% heat-inactivated fetal calf serum (FCS) and penicillin-streptomycin. The cells were grown at 37°C in a 5% CO_2_ environment. For live-cell imaging, cells were seeded onto glass-bottom dishes (#3970-035, AGC TECHNO GLASS CO., LTD., Haibara-gun, Shizuoka, Japan) in live-cell imaging medium, which consisted of phenol red-free EMEM (#05901, Nisssui Pharmaceutical Co., LTD., Tokyo, Japan) supplemented with 5% heat-inactivated FCS, 2 mM L-glutamine, and 2.2 g/L sodium hydrogen carbonate. Vero cell lines stably expressing AcGFP1-Mito were established as described elsewhere (Shimasaki *et al.*, 2024).

### Microscopic observation

To label lipid droplets, cells were incubated for 30 minutes in a live-cell imaging medium containing 1 μM Lipidye II (LD II; FDV-0027, Funakoshi, Tokyo, Japan). For carbonyl cyanide *m*-chlorophenyl hydrazone (CCCP) treatment, cells were incubated in a live-cell imaging medium containing 20 μM CCCP for 4 hours. For oleic acid (OA) treatment, cells were incubated in a live-cell imaging medium containing 500 μM OA/0.5% BSA for 24 hours in the presence of LD II.

The components of the APC microscopy system and conditions for live-cell imaging were described previously (Shimasaki *et al.*, 2024). Briefly, an inverted microscope (ECLIPSE Ti2; MEA54000, Nikon, Tokyo, Japan) equipped with a 100 × objective lens with an apodized phase plate (Plan Fluor ADH 100×/NA 1.30 Oil; MRH41902, Nikon), a white high-power light-emitting diode (LED) (TI2-D-LHLED, Nikon) as a transillumination light source with a narrow bandpass filter (FF01-546/6-45-D, Semrock, Rochester, NY, USA), a multi-wavelength LED illumination system (X-Cite TURBO, Excelitas Technology Corp., Waltham, MA, USA) for epi-fluorescence illumination, and a back-illuminated scientific Complementary Metal-Oxide-Semiconductor (sCMOS) camera (ORCA-Fusion BT, Hamamatsu Photonics K.K., Hamamatsu-shi, Shizuoka, Japan) for image recording. Image acquisition was controlled with NIS-Elements AR software (MQS31000, Nikon). To detect the fluorescent signals of AcGFP1 and LD II, 475 and 430 nm LED lights were used, respectively, with a fluorescent filter cube, GFP Basic C-FL (MXK38474, Nikon). To capture APC and fluorescent images in living cells near-simultaneously (time lag: 400–500 milliseconds), the filter cube was kept in place even during transmitted illumination. All images were recorded and exported as 16-bit ND2 files (2304 × 2304 pixels). All images in each dataset were acquired in at least two independent experiments. For visualization, the contrast of each image of the same condition was adjusted to the same setting based on a look-up table using the ‘Enhance Contrast’ of Fiji software (Schindelin *et al.*, 2012).

### Preprocessing of image dataset for DL

The exported ND2 image files were converted to 16-bit grayscale TIFF files using Fiji and subsequently processed as follows. APC images underwent background subtraction as described previously (Shimasaki *et al.*, 2024). Subtraction of the APC images was carried out in 32-bit float, and the outputs were converted to 8-bit images. Fluorescent images were deconvoluted using NIS-A 2D/3D deconvolution modules (Richardson-Lucy method; MQS42700, Nikon) loaded into the NIS-Elements software. To further enhance the signal-to-noise ratios while maintaining the images less grainy, the following preprocessing procedures were performed using Fiji in AcGFP1-Mito or LD II fluorescent images: (i) Rolling ball background subtraction (Rolling ball radius: 15 pixels for AcGFP1-Mito, 50 pixels for LD II images), (ii) Unsharp Mask (Radius: 5 pixels, Mask Weight: 0.8), and (iii) Gaussian Blur (Sigma: 0.7).

### Application of interactive ML tool, ilastik for generating ground truth masks

To generate ground truth mask images from the preprocessed fluorescent images, we utilized an interactive ML tool, ilastik version 1.4.0 (Berg *et al.*, 2019) (https://github.com/ilastik). By manually annotating pixels as foreground or background classes, the classifier was trained to assign each pixel to a class based on image features such as intensity, edge, and texture. The ilastik pipelines are described as follows:

*0. Creating New Project*: Select the workflow of pixel classification + object classification.

*1. Input Data*: Load the dataset for ilastik classifier training.

*2. Feature Selection*: Select all 37 features in the default settings.

*3. Training (Pixel Classification)*: Annotate pixels for the foreground (class 1) and background (class 2).

*4. Thresholding*: Select the following parameters; For AcGFP1-Mito, *Method*: Simple, *Input*: 0 (Foreground), *Smoothing Sigma*: 1.0 (X dimension) and 1.0 (Y dimension), *Threshold Value*: 0.5, *Size Filter*: 10 (Min)–1000000 (Max). For LD II, *Method*: Simple, *Input*: 0 (Foreground), *Smoothing Sigma*: 1.0 (X dimension) and 1.0 (Y dimension), *Threshold Value*: 0.4 (OA-untreated) or 0.5 (OA-treated), *Size Filter*: 10 (Min)–1000000 (Max).

*5. Object Feature Selection*: Select 35 features, excluding location ones. Neighborhood size in pixels is 30 (X) and 30 (Y).

*6. Object Classification*: Label classes as targeted objects (class 1) and debris (class 2)

*7. Object Information Export*: Select “Object Predictions” for exporting PNG files

The labels annotated in the pixel classification and object classification sections are provided in the Supplemental Materials.

### Application of deepflash2 for deep learning procedures

We utilized deepflash2 version 0.2.3, an out-of-the-box tool providing a pipeline for training, evaluation, and application of deep learning models (Griebel *et al.*, 2023) (https://github.com/matjesg/deepflash2). The DL models for semantic segmentation were trained using ConvNeXt, an advanced convolution neural network architecture inspired by transformer models to enhance performance and efficiency (Liu *et al.*, 2022). The loss function for DL training was CrossEntropyDiceLoss. The model ensemble was generated from five models. The hyperparameters set in the graphical user interface are described as follows:

Training Parameters; *Train Epochs*: 30, *Learning Rate*: 0.001 for mitochondria and 0.0005 for lipid droplets, *Model Architecture*: Unet, *Encoder*: tu-convnext_base_384_in22ft1k, *Encoder Weights*: imagenet, *Mini-Batch Size*: 4, *Mixed Precision Training*: Yes, *Weight Decay*: 0.001 for mitochondria and 0.0005 for lipid droplets, *Optimizer*: RAdam, *Sample Multiplier*: 0, *Scale factor*: 1

Data Augmentation; *Flip*: Yes, *Rotation*: 360, *Gamma (lower limit)*: 80, *Gamma (upper limit)*: 120, *Brightness (limit)*: 0, *Contrast (limit)*: 0, *CLAHE (clip limit)*: 0, *GridDistortion (limit)*: 0

To prevent potential overfitting during the training phase, deepflash2 displays the training loss and validation loss of models at each epoch. We monitored these loss values and stopped training at the epoch where both values were declining in tandem and were close to each other. The training curves of these losses over epochs during training of DL models are presented in Supplemental Fig. 2.

For validating the accuracy of predicted results, deepflash2 used the Dice score (Zou *et al.*, 2004), a metric calculated as follows:


$$Dice\;score=\frac{TP}{TP+\frac{1}{2}(FP+FN)}\;$$

where true positives (TP) indicate the sum of the areas overlapping between the predicted and ground truth masks, and false positives (FP) and false negatives (FN) indicate the areas present only in the predicted or the ground truth masks, respectively.

The training and validation datasets, and the loss functions at each epoch during the training of models are provided in Supplemental Materials.

### CellProfiler pipelines for morphological analysis

We utilized CellProfier version 4.2.6 for automated morphological analysis (Stirling *et al.*, 2021). The cell mask images were manually generated from APC images using Fiji. For measuring mitochondrial length per cell (Fig. 3E), the image analysis was performed as the follows in CellProfiler pipeline:

*0. Images*: Load image sets of the cell masks, ground truth masks, and predicted results from the same images.

*1. MaskImage*: Crop the images of the ground truth and predicted masks using cell mask images.

*2. MorphologicalSkeleton*: Skeltonize the mitochondrial mask images.

*3. ConvertImageToObjects*: Convert the attribution of masked images (produced in steps 1 and 2) from “image” to “objects”.

*4. MeasureObjectSizeShape*: Measure the areas of the cell masks and skeletonized objects (produced in step 3).

*5. RelateObjects*: Associate the skeletonized objects with their parent cell masks.

*6. ExportToSpreadsheet*: Export the results of the mean values per cell as CSV files.

For measuring the areas of lipid droplets per cell (Fig. 3J), the image analysis was carried out as follows in the CellProfiler pipeline:

*0. Images*: Load image sets of the cell masks, ground truth masks, and predicted results from the same images.

*1. MaskImage*: Crop the images of the ground truth and predicted masks using cell mask images.

*2. ConvertImageToObjects*: Convert the attribution of masked images (produced in step 1) from “image” to “objects”.

*3. RelateObjects*: Associate the lipid droplet masks with their parent cell masks.

*4. SplitOrMergeObjects*: Merge the individual lipid droplet masks into one object.

*5. RelateObjects*: Associate the merged objects (produced in step 4) with their parent cell masks.

*6. MeasureObjectsSizeShape*: Measure the areas of the cell masks and merged objects (produced in step 4).

*7. CalculateMath*: Calculate the ratios of the areas of object masks to those of cell masks.

*8. ExportToSpreadsheet*: Export the results of the mean values per cell as CSV files.

### Statistical analysis

Statistical analyses were performed using EZR software (Kanda, 2013), which is a graphical user interface for R (The R Foundation for Statistical Computing, Vienna, Austria). The Mann-Whitney U test was carried out for two-group comparisons. *P*-values of 0.001 or less were considered statistically significant.

## Results and Discussions

**Fig. 1:**
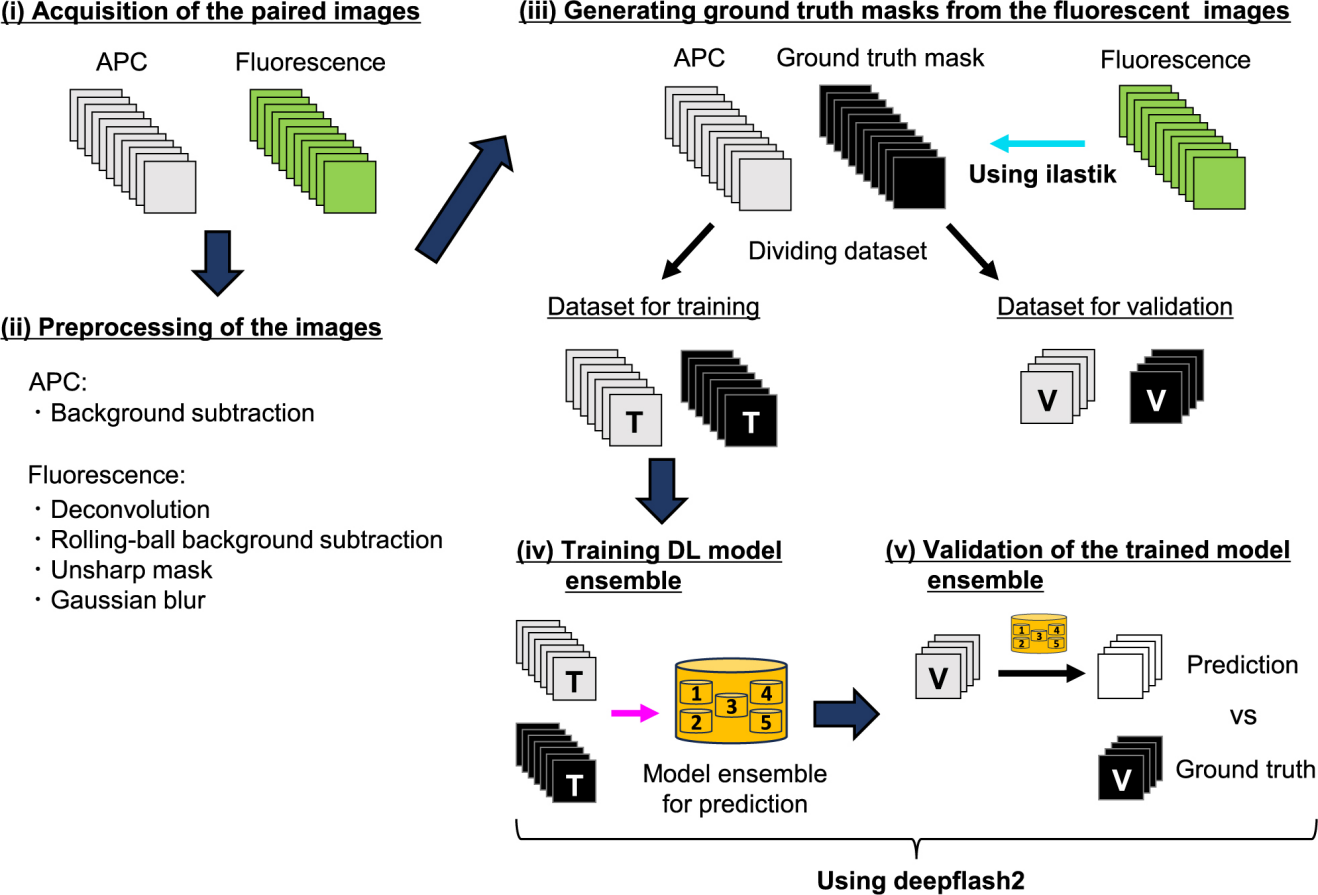
A schematic workflow depicting the establishment of DL-based segmentation models for subcellular organelles in APC images. (i) Living cells labeled with fluorescent markers of targeted objects were imaged, acquiring paired APC and fluorescent images near-simultaneously. (ii) Image processing was performed using Fiji software following the indicated methods. (iii) Ground truth mask images were generated from preprocessed fluorescent images using a classifier trained with an interactive ML-based approach in ilastik software. These paired images were divided into two datasets: one for training DL model ensemble for prediction (referred to as ‘T’) and the other for validating the trained models (referred to as ‘V’). (iv) DL model ensemble for predicting targeted objects in APC images was trained using deepflash2. (v) The trained model ensemble was validated by comparing predicted segmentation results with ground truth masks in the validation dataset, using criteria such as dice scores.

A schematic workflow illustrating the development of DL-based segmentation models is presented in Fig. 1. In stage (i), we imaged living cells labeled with fluorescent markers of targeted organelles, acquiring paired APC and fluorescent images near-simultaneously. These paired images were subjected to preprocessing in stage (ii) to enhance the signal-to-noise ratios while maintaining the images with less grainy. For effective supervised training of segmentation models, it is crucial to utilize paired images of targeted objects alongside accurate ground truth binary masks, as inaccurate masks can negatively impact model training (Segebarth *et al.*, 2020; Von Chamier *et al.*, 2021). In stage (iii), to generate more precise ground truth masks from preprocessed fluorescent images, we utilized “ilastik” software (Berg *et al.*, 2019), which facilitates training pixel classifiers with interactive ML based on image features such as local intensity, edge, and texture. The trained classifier yielded binary masks that were more informative than those produced by conventional thresholding approaches (Supplemental Fig. 1). In regions with higher signal intensities, fluorescence was converted to binary masks with acceptable accuracy using both methods (yellow arrows in ROI 1: Supplemental Fig. 1). However, Otsu’s method failed to separate areas with lower signal intensities (magenta arrows in ROI 1 and the area of ROI 2: Supplemental Fig. 1). This discrepancy likely arises because Otsu’s method determined the intensity threshold based on the histogram of the entire image, failing to detect local variations in image features.

In the final step in stage (iii), the obtained paired APC and ground truth mask images were divided into two datasets: one for training DL models and the other for validating the trained models. In stage (iv), to achieve training DL model ensemble, we employed “deepflash2”, a tool offering a pipeline for training, evaluation, and application of deep learning models with a user-friendly graphical interface, suitable for researchers without programming skills (Griebel *et al.*, 2023). Additionally, deepflash2 implements model ensemble techniques to ensure higher accuracy and reproducibility compared to a single DL model (Ganaie *et al.*, 2022).

### Validation of the trained DL models for segmentation of organelles in APC images

**Fig. 2:**
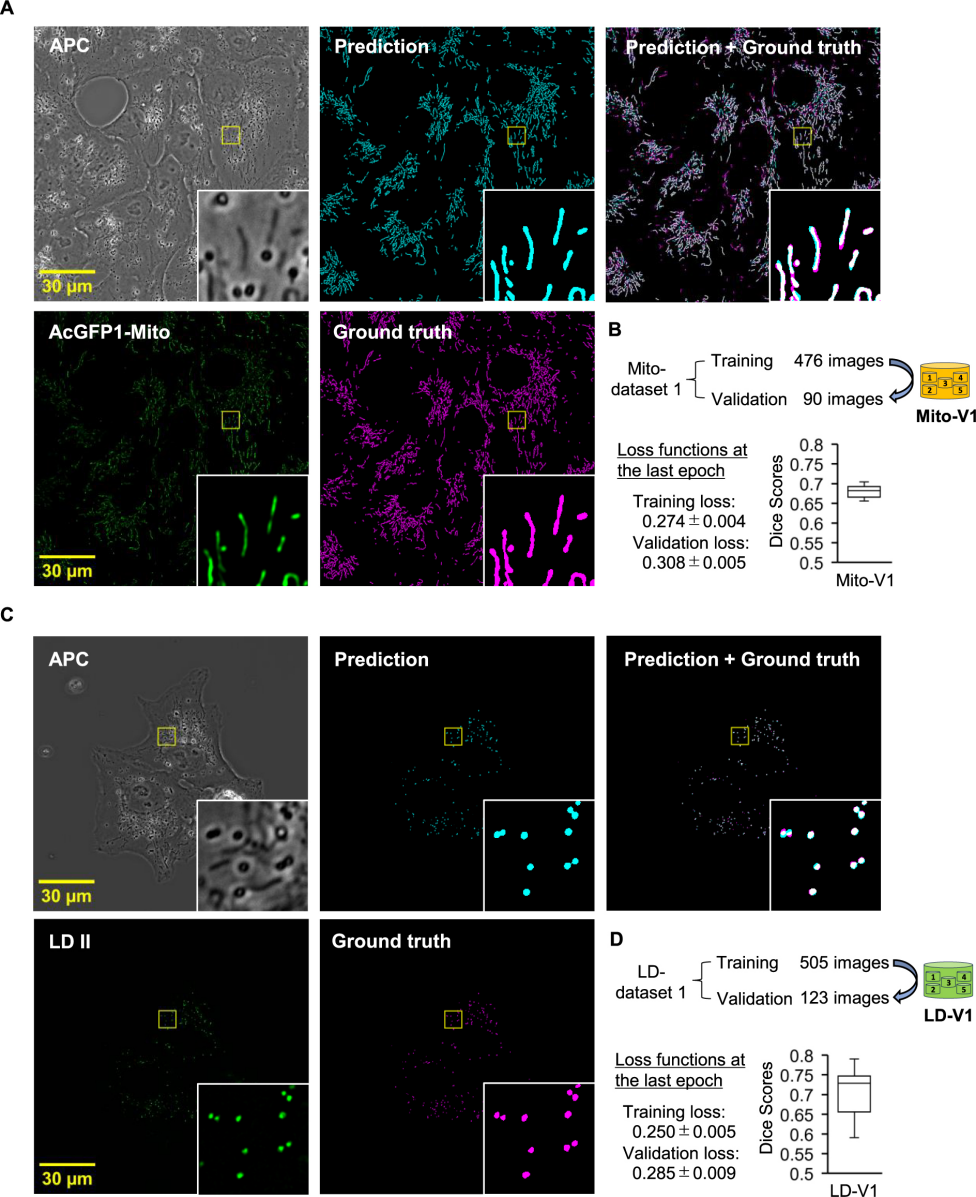
Validation of the trained DL model ensembles for predicting organelles in APC images. (A, C) APC and fluorescent images of Vero cells expressing AcGFP1-Mito (A), or labeled with LD II (C), were utilized for DL model ensemble training. Representative images show APC, fluorescent markers (green), ground truth mask (magenta), and prediction (cyan). Insets magnify areas corresponding to yellow boxes in each image. Scale bars are 30 μm. (B, D) Accuracy of the trained model ensemble was validated using Dice scores for mitochondria (B, n = 90 images), and lipid droplets (D, n = 123 images). Mito-V1 and LD-V1 were trained using datasets containing 476 and 505 images, respectively, for segmentation of each organelle. The values of training and validation loss functions at the last epoch during the training of models are shown as means ± SD (from 5 models). The learning curves of the losses over epochs during training for Mito-V1 and LD-V1 are presented in Supplemental Fig. 2A and B. Box plots show dice scores per image in the validation dataset, with the median indicated by the line inside the box. The bottom and top of the box represent the first and third quartiles, respectively. Whiskers extend from the box to the minimum and maximum values of the data.

Through the DL-based segmentation workflow, we developed trained DL model ensembles for predicting mitochondria and lipid droplets in APC images (Fig. 2A and C). Comparing the training and validation loss functions of the models indicated that potential overfitting could be avoided, as the values of each loss were close (Fig. 2B, D, and Supplemental Fig. 2A, B). The trained models were In stage (v) of our workflow (Fig. 1), we validated these ensembles, referred to as Mito-V1 for mitochondria and LD-V1 for lipid droplets, by quantitatively evaluating the predicted results using Dice scores. The Dice score is a widely used criterion for measuring the accuracy of image segmentation by comparing the overlap between predicted and ground truth segmentation (Zou *et al.*, 2004). This metric ranges from 0 to 1, where 1 indicates perfect agreement and 0 indicates no overlap between the two segmentation images, with higher scores reflecting better performance. The Dice scores for the Mito-V1 and LD-V1 were 0.680 ± 0.014 (n = 90 images) and 0.708 ± 0.052 (n = 123 images), respectively (Fig. 2B and D).

For further validation, we visually inspected APC, ground truth, and predicted images (Supplemental Fig. 3). It is noteworthy that although our APC microscopy system can capture the APC and fluorescent images near-simultaneously, there is a time lag between these images due to the switching of the light source and the exposure time for image recording (Supplemental Fig. 3A). This time lag can cause significant spatiotemporal discrepancies between the two images, particularly for objects that move actively during this time lag. In Supplemental Fig. 3B, the predicted results for objects A and B appeared acceptable; however, object A had a lower Dice score due to movement during the time lag, leading to location inconsistencies in APC and fluorescent images, and, consequently, discrepancies between the predicted and ground truth masks. These findings suggest that while Dice scores are valuable for assessing segmentation accuracy in APC images, additional evaluations should be performed to determine whether the model ensembles are practically applicable in bioimage analysis.

### Practical evaluation of DL-based segmentation models for the organelle’s morphological analysis

**Fig. 3:**
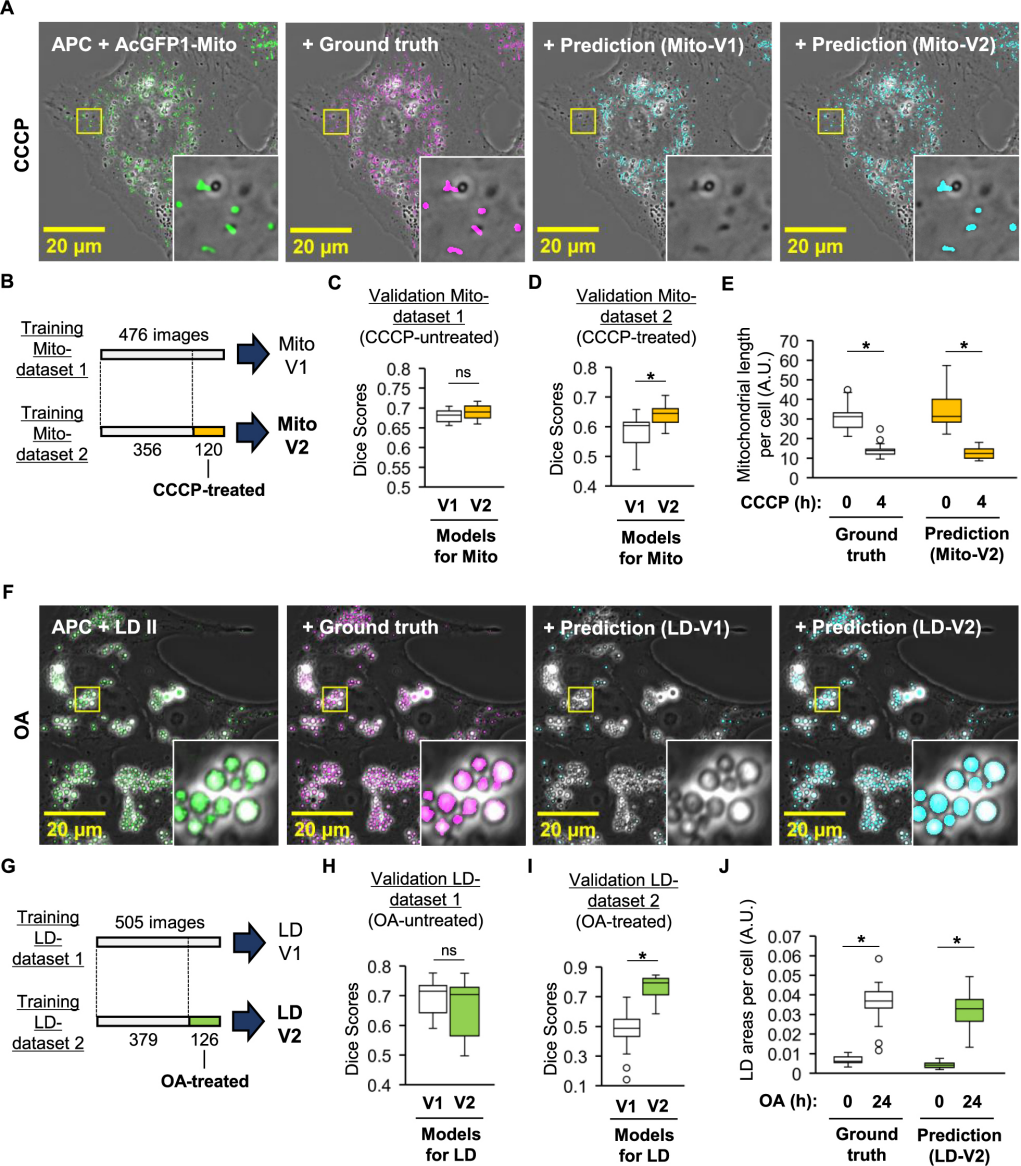
Practical assessment of DL-based segmentation results in terms of the organelle’s morphological analysis. (A) Vero cells expressing AcGFP1-Mito were treated with 20 μM CCCP for 4 hours. Representative images show APC merged with AcGFP1-Mito (green), ground truth mask (magenta), and predicted results by Mito-V1 (cyan), or Mito-V2 (cyan). Insets magnify areas corresponding to yellow boxes in each image. (B) Training Mito-dataset 1 for predicting mitochondria, identical to that in Fig. 2B, consists of 476 images from untreated cells. Training Mito-dataset 2 was generated by replacing 120 images from training dataset 1 with 120 images from CCCP-treated cells. The resulting DL model ensemble trained on Mito-dataset 2 is denoted as Mito-V2. The loss function values during the training of 5 models for Mito-V2 ensemble at the last epoch were 0.305 ± 0.006 (mean ± SD) for training loss and 0.348 ± 0.006 for validation loss, respectively. The learning curves of the losses over epochs during training for Mito-V2 are shown in Supplemental Fig. 2C. (C) Validation of Mito-V2 model ensemble for predicting mitochondria in CCCP-untreated cells using validation Mito-dataset 1 (n = 90 images), consistent with Fig. 2B. The box plot depicts dice scores for each image. (D) Validation of Mito-V1 and Mito-V2 for predicting mitochondria in CCCP-treated cells using validation Mito- dataset 2 (n = 23 images). (E) Analysis of mitochondrial morphological alterations induced by CCCP compared to results from ground truth masks and predictions by Mito-V2. Images at 0 and 4 hours post-CCCP treatment were analyzed (n = 23 and 25 cells at 0 and 4 hours, respectively). The box plots show the means of mitochondrial lengths per cell under each condition. The dots represent data outside the whiskers. (F) Vero cells labeled with LD II were treated with 500 μM OA for 24 hours. Representative images show APC merged with LD II (green), ground truth mask (magenta), and predicted results by LD-V1 (cyan), and LD-V2 (cyan). (G) Training LD-dataset 1 for predicting lipid droplets, identical to that in Fig. 2D, consists of 505 images from untreated cells. Training LD-dataset 2 was generated by replacing 126 images from training dataset 1 with 126 images from OA-treated cells. The resulting DL model ensemble trained on dataset 2 is denoted as LD-V2. The loss function values during the training of 5 models for LD-V2 ensemble at the last epoch were 0.237 ± 0.006 (mean ± SD) for training loss and 0.282 ± 0.007 for validation loss, respectively. The learning curves of the losses over epochs during training for LD-V2 are presented in Supplemental Fig. 2D. (H) Validation of LD-V2 for predicting lipid droplets in OA-untreated cells using validation LD- dataset 1 (n = 123 images), consistent with Fig. 2D. The box plots show dice scores for each image. (I) Validation of LD-V1 and LD-V2 for predicting lipid droplets in OA-treated cells using validation LD-dataset 2 (n = 31 images). (J) Analysis of morphological alterations of lipid droplets induced by OA compared to results from ground truth masks and predictions by LD-V2. Images at 0 and 24 hours post-OA treatment were analyzed (n = 13 and 17 cells at 0 and 24 hours, respectively). The box plots indicate the means of the ratios of LD area to whole cell area per cell. *: *p*<0.001, ns: not significant (Mann-Whitney U test). Scale bars in (A) and (F): 20 μm.

To evaluate the practical applicability of the trained DL model ensembles, we tested their ability to detect morphological alterations of organelles in APC images. We treated AcGFP1-Mito-expressing Vero cells with CCCP, a mitochondrial membrane potential erasing agent, to induce mitochondrial fragmentation (Ishihara *et al.*, 2003). While CCCP-treated cells exhibited fragmented mitochondria in fluorescent and ground truth mask images, Mito-V1 seemingly failed to detect these fragmented mitochondria (Fig. 3A). This failure may be attributed to Mito-V1 having been based on training Mito-dataset 1, which consisted of images from untreated cells, making it less capable of detecting fragmented mitochondria. To enhance the robustness of the DL model ensemble, we created a new training dataset, Mito-dataset 2, by replacing approximately 25% of the images in training Mito-dataset 1 with images from CCCP-treated cells. We then trained models that could avoid the potential overfitting (Training loss: 0.305 ± 0.006, Validation loss: 0.348 ± 0.006), resulting in a new DL model ensemble, Mito-V2 (Fig. 3B and Supplemental Fig. 2C). The resulting Mito-V2 predicted mitochondria with similar accuracy to Mito-V1 in untreated cells (Fig. 3C). Additionally, Mito-V2 demonstrated higher accuracy in predicting mitochondria in CCCP-treated cells than Mito-V1 (Fig. 3D), indicating successful robustness enhancement. Morphological analysis of mitochondrial length using Mito-V2’s predictions showed decreases in length consistent with ground truth mask results (Fig. 3E).

To further assess the DL model ensemble’s ability to predict lipid droplets, we performed similar experiments in the case of mitochondria. We treated LD II-labeled cells with OA to induce lipid droplet accumulation (Nakamura *et al.*, 2005). OA-treated cells showed a noticeable increase in lipid droplets in fluorescent and ground truth mask images, but LD-V1 failed to predict these accumulated objects (Fig. 3F). We trained DL models that could avoid overfitting (Training loss: 0.237 ± 0.006, Validation loss: 0.282 ± 0.007), resulting in a new DL model ensemble, LD-V2, using training LD-dataset 2, which included images from OA-treated cells (Fig. 3G and Supplemental Fig. 2D). LD-V2 predicted lipid droplets with similar accuracy to LD-V1 in the OA-untreated validation LD-dataset 1 (Fig. 3H). Moreover, LD-V2 demonstrated higher accuracy in predicting lipid droplets in OA-treated cells than LD-V1 (Fig. 3I). Morphological analysis indicated that increases in lipid droplet mass per cell in response to OA treatment were detected in both predicted and ground truth mask results (Fig. 3J). Taken together, these results indicate that the segmentation results predicted by the DL model ensembles are practically acceptable for morphological analysis.

To enhance the prediction accuracy of DL model ensembles, several approaches can be considered in at least two aspects: microscopic and computational. In the microscopic aspect, utilizing an objective lens with a higher numerical aperture (NA) can enhance the image resolution of both phase contrast and fluorescence, which may positively impact prediction accuracy, particularly for smaller structures that appear faint in APC images. However, it is also possible that, for certain objects, lower-resolution images might be more suitable for DL to learn these features. Integrating a high NA objective into the APC microscopy system and acquiring phase-contrast images requires an external phase plate setup, as high NA objectives often do not have an internal phase plate. Externally placing a phase plate outside the objective, in an optically relayed plane conjugate to the objective back focal plane, allows for phase-contrast image recording using objectives without an internal phase plate (Pelc *et al.*, 2020). Additionally, high NA objectives can shorten exposure times for image recording, minimizing spatiotemporal discrepancies between the phase-contrast and fluorescent images, and thus correcting discrepancies in Dice scores (Supplemental Fig. 3). Another approach to overcoming these discrepancies is integrating a two-camera system, which allows for the simultaneous capture of two types of images, thereby eliminating the time lag between phase-contrast and fluorescent images.

In the computational aspect, increasing the number of images in the training dataset may enhance the accuracy of DL model ensembles. Adding images from CCCP- or OA-treated cells to the training dataset has been shown to enhance the robustness of DL model ensembles (Fig. 3). These results suggest that preparing training datasets comprising images from various culture conditions is important for training robust DL models. Using deepflash2, we utilized the base variant of the ConvNeXt architecture for DL model training (Liu *et al.*, 2022). Leveraging deeper and more complex models, such as the large or xlarge variants, may achieve better performance at the expense of computational resources. Another approach to enhancing accuracy is utilizing more powerful DL architectures, such as ConvNeXt2 (Woo *et al.*, 2023), although this is not supported by the latest version of deepflash2 (v2.4.0).

For establishing DL models to predict other organelles, using highly specific fluorescent markers is necessary. In our previous study (Shimasaki *et al.*, 2024), we labeled lysosomes with LysoTracker Green (LTG) to determine the visual characteristics of lysosomes in the APC images and observed that most lysosomes appeared as darker vesicles than the cytosol. Although this probe is widely used as a fluorescent marker for lysosomes, it can also accumulate in and stain other acidic compartments such as late endosomes. Therefore, the ground truth mask images generated from the LTG-labeled fluorescent images may contain labels for both lysosomes and other acidic compartments. Even a small portion of label contamination in the ground truth mask images can lead to the misrepresentation of other objects with different visual characteristics as lysosomes, thereby hindering the training of highly accurate DL models. Regarding AcGFP1-RhoB used as an endosomal marker in our previous study, we also consider it unsuitable, particularly for training accurate DL models due to its specificity issues (Castillo *et al.*, 2023). To establish segmentation models for organelles in the endosomes/lysosomes pathway, further analysis is needed to explore the transition of the visual characteristics from early endosomes to late endosomes and lysosomes in the APC images using highly specific markers such as EEA1 (Mu *et al.*, 1995), Rab7 (Borchers *et al.*, 2021), and LAMP1/2 (Chen *et al.*, 1985).

## Conclusions

By leveraging AI technologies, including interactive ML and DL, with the APC microscopy system, we established a comprehensive workflow for training segmentation models for subcellular organelles in APC images. The evaluation of trained DL model ensembles in morphological analysis indicated their effectiveness in predicting mitochondria and lipid droplets with practically applicable accuracy, providing a robust framework for studying organelle dynamics in unstained living cells.

Applying the DL-based segmentation workflow to various organelles can generate predicted masks on these structures from APC images. These masks can be utilized for morphological analysis using image features such as area, length, circularity, object numbers per cell, and proximities between organelles. This quantitative information from a single APC image has the potential not only to study organelle dynamics but also to provide criteria for drug screening or assessing cellular homeostasis.

It is noteworthy that predicted masks from the DL models for distinct organelles presented in this workflow are not suitable for colocalization analysis. Overlapping masks predicted by distinct semantic segmentation models in a single image indicate misprediction of pixels rather than true colocalization of two separate objects.

## Data Availability

We deposited the raw and processed images, analyzed results for figure preparation, and the dataset for establishing organelle segmentation models in J-STAGE Data (doi: 10.51021/data.csf.26377540).

## Funding

This work was financially supported in part by MEXT KAKENHI (No. JP17H06417), JSPS KAKENHI (JP23K21362), and AMED (JP22fk0108561j0101) to K.H.; MEXT KAKENHI (No. JP17H06413) to K.K.; AMED (JP20he0622012) to K.K. and K.H.; Morinomiyako Medical Research Foundation to K.Sh.

## Data Availability

The supporting information for this article is available in J-STAGE Data.

## Abbreviations

AI: artificial intelligence
APC: apodized phase contrast
CCCP: carbonyl cyanide *m*-chlorophenyl hydrazone
DL: deep learning
FN: false negatives
FP: false positives
LD II: LipiDye II
LED: light-emitting diode
LTG: LysoTracker Green
ML: machine learning
NA: numerical aperture
OA: oleic acids
ROI: region of interest
sCMOS: scientific Complementary Metal-Oxide-Semiconductor
TP: true positives
