# Supplementary material for: Deep learning-based segmentation of subcellular organelles in high-resolution phase-contrast images
Source: Cell Struct Funct. 2024 Jul 31;49(2):57–65. doi: 10.1247/csf.24036 (PMC11930775; doi:10.1247/csf.24036)
Supplement: Supplementary file 1 — Supplementary Materials [file csf_49_24036_1.pdf]

Supplemental Figure 1.

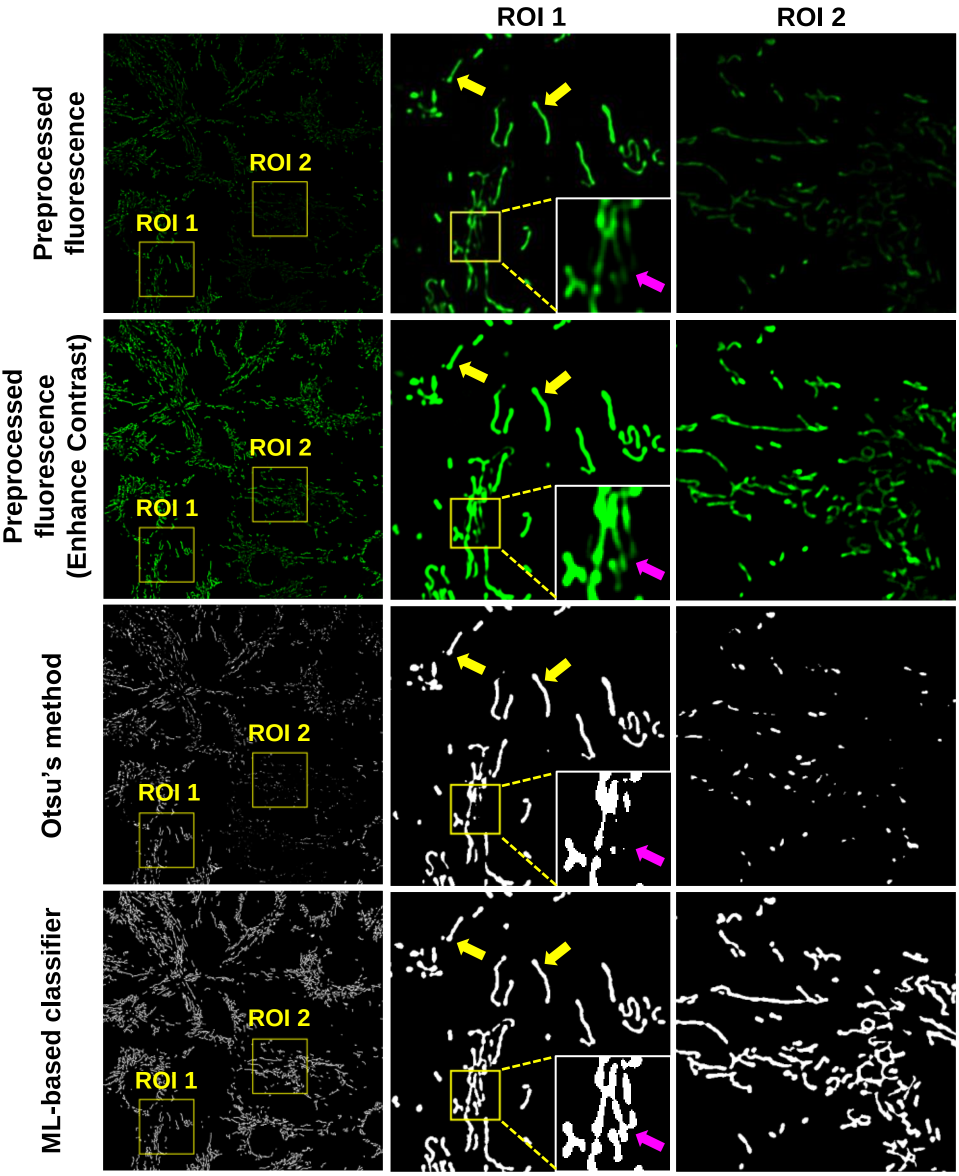

Supplemental Figure 2.

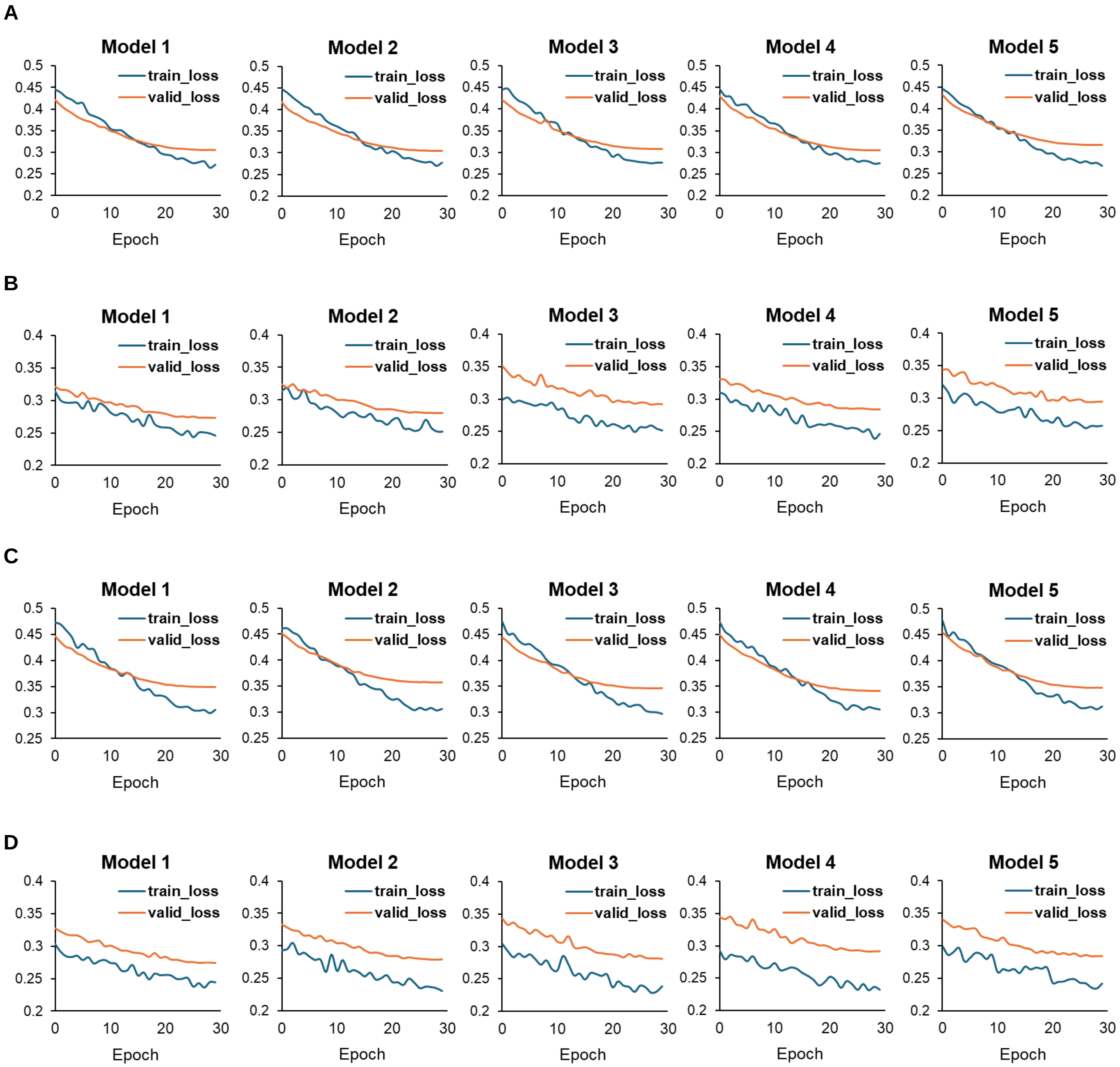

Supplemental Figure 3.

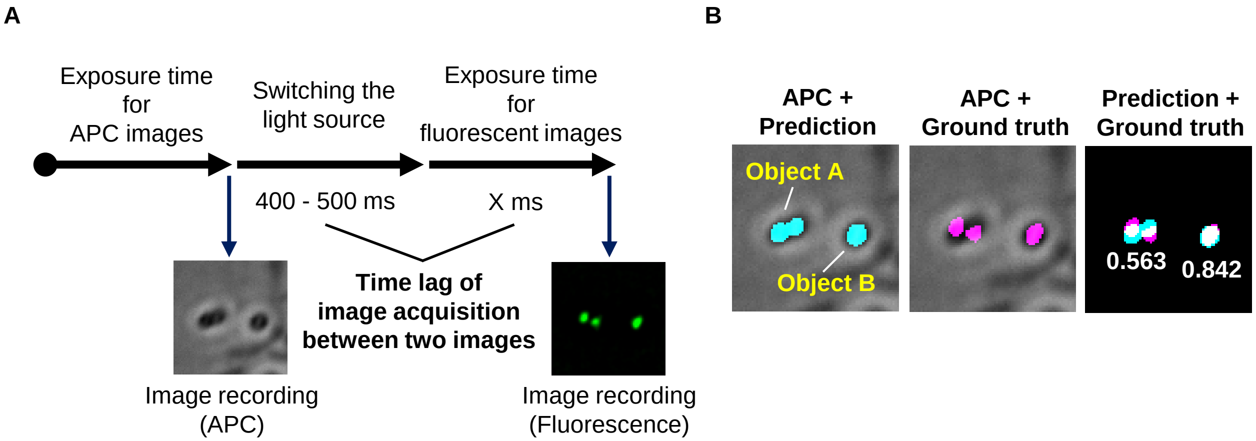

Supplemental Figure Legends.

Supplemental Fig 1. The efficiency of interactive ML-based pixel classifiers for generating ground truth masks from fluorescent organelle marker images. Representative images show Vero cells expressing AcGFP1-Mito. Preprocessed fluorescence images (without contrast enhancement) were converted to binary images using conventional thresholding approaches such as Otsu's method (using Fiji) or ML-based classifiers trained using ilastik. Both approaches successfully separated areas with higher signal intensities (yellow arrows in ROI 1). However, Otsu's method failed to separate areas with lower intensities (magenta arrows in ROI 1 and ROI 2).

Supplemental Fig 2. The learning curves during training of each DL model. The plots of the loss function values over epochs during training models from Mito-V1 (A), LD-V1 (B), Mito-V2 (C), and LD-V2 (D) ensembles are shown. The values of each plot are presented in deposited data at J-Stage Data.

Supplemental Fig 3. Detailed validation of predicted segmentation results. (A) The time lag between APC and fluorescent image acquisition in the APC microscopy system includes the time for switching light sources (400 - 500 milliseconds) and varying exposure times for image recording under different conditions. (B) Representative images depict APC merged with predictions (cyan) and ground truth masks (magenta) in a cell labeled with LD II. Dice scores for objects A and B (0.563 and 0.842, respectively) are indicated in the merged images of predicted results and ground truth masks.
